# Comparative pharmacokinetics and safety evaluation of high dosage regimens of *Andrographis paniculata* aqueous extract after single and multiple oral administration in healthy participants

**DOI:** 10.3389/fphar.2023.1230401

**Published:** 2023-08-17

**Authors:** Phanit Songvut, Nuchanart Rangkadilok, Nanthanit Pholphana, Tawit Suriyo, Duangchit Panomvana, Porranee Puranajoti, Jaratluck Akanimanee, Jutamaad Satayavivad

**Affiliations:** ^1^ Laboratory of Pharmacology, Chulabhorn Research Institute, Bangkok, Thailand; ^2^ Center of Excellence on Environmental Health and Toxicology (EHT), OPS, MHESI, Bangkok, Thailand; ^3^ Translational Research Unit, Chulabhorn Research Institute, Bangkok, Thailand

**Keywords:** high dose of andrographolide, pharmacokinetics, *Andrographis paniculata*, aqueous extract, safety evaluation

## Abstract

**Background:** The prolonged situation of the COVID-19 pandemic, with the emergence of new variants of SARS-CoV-2, not only imposes a financial burden on healthcare supports but also contributes to the issue of medication shortages, particularly in countries with limited access to medical resources or developing countries. To provide an alternative therapeutic approach during this crisis, there is an increasing research that has investigated the potential uses of *Andrographis paniculata* in supporting the application of herbal medicine for COVID-19.

**Purpose:** This study aimed to investigate the safety profiles and clinical pharmacokinetics, specifically focusing on dose proportionality of the four major active diterpenoids of *Andrographis paniculata* aqueous extract following oral administration of two different high doses of andrographolide.

**Methods:** The participants received the aqueous extract capsules equivalent to 60 or 120 mg of andrographolide; and as multiple doses administered three times daily, calculated as 180 or 360 mg/day of andrographolide. Safety evaluation was assessed following the oral administration of the multiple doses.

**Results:** The results indicated a dose-dependent effect observed between the respective two doses. A twofold increase in the dose of the extract demonstrated twofold higher plasma concentrations of the four major parent compounds; 1) andrographolide, 2) 14-deoxy-11, 12-didehydroandrographolide, 3) neoandrographolide, and 4) 14-deoxyandrographolide, as well as their conjugated metabolites. The observed diterpenoids are biotransformed partly through a phase II metabolic pathway of conjugation, thus reducing in the parent compounds in the plasma and existing the majority as conjugated metabolites. These metabolites are then excreted through the hepatobiliary system and urinary elimination. For the results of the safety evaluation, the occasional adverse events experienced by individuals were of mild intensity, infrequent in occurrence, and reversible to the normal baseline. Safety consideration should be given to the individual patient’s pertinent health conditions when using this extract in patients with hepatic or kidney dysfunction.

**Clinical Trial Registration:** https://www.thaiclinicaltrials.org/show/TCTR20210201005; Identifier: TCTR20210201005.

## 1 Introduction

Coronavirus disease (COVID-19) pandemic is caused by the severe acute respiratory syndrome coronavirus 2 (SARS-CoV-2). It has been an epidemic that resulted in a significant number of fatalities in many countries. However, a current statement of WHO from the 15th meeting (on 4th May 2023) of the International Health Regulations (IHR) Emergency Committee on the COVID-19 pandemic concluded that COVID-19 is now an established and ongoing health issue which no longer constitutes a public health emergency of international concern (PHEIC) (WHO, 2023). Nonetheless, it continues to present the intricate public health challenges. Researchers have been exploring potential treatments and supportive care options for the disease during the outbreaks. The use of preventive vaccines and antiviral drugs is recommended for managing COVID-19; however, there are reports of adverse effects and limited efficacy associated with these uses. Moreover, in developing countries, the limited availability of commercial antiviral drugs, including molnupiravir, favipiravir or remdesivir, contributes to the ineffective management of COVID-19. The extended duration of COVID-19 treatment also imposes a financial burden on healthcare systems. The emergence of new variants of SARS-CoV-2 has further intensified the issue of medication shortages in countries with limited access to medical resources.

Extensive research has been conducted to support an alternative therapeutic approach for the COVID-19 crisis, focusing on the potential benefits of *Andrographis paniculata* (Burm. f.) Nees. This phytomedicine has traditionally been used in the treatment of upper respiratory tract infections (URTIs) and is now being investigated as an option for COVID-19 patients in Thailand, China, India, and other Asian countries. In Thailand, the oral administration of *Andrographis paniculata* equivalent to 180 mg/day of andrographolide significantly reduced the incidence of pneumonia among nonimmune patients with early-stage COVID-19 (Benjaponpitak et al., 2023). In China, the use of *A. paniculata* in patients with mild to moderate COVID-19 symptoms, who received standard supportive care along with a water-soluble andrographolide sulfonate administered intravenously at a dosage of 10 mg/kg/day (maximum 500 mg/day) for 7–14 days, demonstrated a decreased probability of progressing to the severe stage of the disease (Zhang et al., 2021). In addition, a fixed combination of herbal medicine (with a daily dose of andrographolide at 90 mg) was studied in Georgia for the treatment of mild and moderate COVID-19 patients and the results demonstrated a significantly reduced disease progression rate compared to the placebo group (Ratiani et al., 2022). Other clinical studies have been conducted to investigate the efficacy of *A. paniculata* in the treatment of viral infectious diseases, including influenza (Kulichenko et al., 2003; Chuthaputti et al., 2007), herpes simplex virus (HSV), human immunodeficiency virus (HIV), coronavirus (SARS-CoV-2) (Zhang et al., 2021; Benjaponpitak et al., 2023), and upper respiratory tract infections (URTIs) (Jayakumar et al., 2013).

In consideration, the majority of andrographolide absorbed into the bloodstream undergoes biotransformations (Songvut et al., 2023), primarily enzymatic processes. However, these processes can become saturated when higher dosages are administered, leading to nonlinear pharmacokinetic behavior. Therefore, in order to use high-dose andrographolide for treatment, it is imperative to conduct a comprehensive study of the pharmacokinetics of *A. paniculata*, with a specific focus on the linear kinetics or dose proportionality. The previous clinical pharmacokinetic studies of *A. paniculata* have focused on the parent compounds (diterpenoids)*,* and have only considered a low dose of 20 mg of andrographolide (Panossian et al., 2000), or multiple doses of 97.92 mg/day of andrographolide (Pholphana et al., 2016). Our pilot study of pharmacokinetics in healthy volunteers involved a single orally administered dose of *A. paniculata* extract (equivalent to 60 mg of andrographolide) (Songvut et al., 2023); however, the information on multiple oral administration of the high dose (60 mg × 3 times/days calculated as 180 mg/day of andrographolide) was still limited, especially in terms of dose proportionality. The objective of this study was to investigate the clinical pharmacokinetics, specifically focusing on dose proportionality, of the four major active diterpenoids 1) andrographolide, 2) 14-deoxy-11, 12-didehydroandrographolide, 3) neoandrographolide, and 4) 14-deoxyandrographolide, after oral administration of two different high doses of andrographolide: a single dose of 60 or 120 mg, and multiple doses of 180 or 360 mg/day. Based on the evidences *A. paniculata*, andrographolide undergo biotransformation in phase II metabolic pathways (Coon and Ernst, 2004; Cui et al., 2005; Ye et al., 2011; Bera et al., 2014; Songvut et al., 2023). This study also includes the investigation of glucuronide and sulfate conjugation of the four parent diterpenoids in plasma and urine by indirect analysis of deconjugation using glucuronidase and sulfatase enzymes.

Taking into account, the research on its aqueous extract, which has a well-established traditional use, has been limited and necessitates further investigation. Therefore, this present study developed an aqueous extract of *A. paniculata* for subsequent determination of its clinical pharmacokinetics. The available information on the safety of the aqueous extract remains limited. Therefore, a secondary objective of this study was to investigate the adverse events that may occur following the oral administration of aqueous extract capsules containing a high content of andrographolide during the pharmacokinetic investigation. The finding of this study on the safety and pharmacokinetic characteristics of high dose of *A. paniculata* aqueous extract offers valuable evidence for considering an appropriate dosage for further studies involving COVID-19 patients.

## 2 Materials and methods

### 2.1 Chemicals

All analytical standards including andrographolide (purity = 100.00%); 14-deoxy-11, 12-didehydroandrographolide (purity = 99.80%); neoandrographolide (purity = 99.67%); and 14-deoxyandrographolide (purity = 100.00%) were purchased from Phytolab GmbH and Co.KG (Vestenbergsgreuth, Germany). The digoxin (purity >95.0%) used as an internal standard (IS) was obtained from Sigma-Aldrich (St. Louis, MO, United States). HPLC-grade acetonitrile and methanol were purchased from Merck (Darmstadt, FR, Germany). The liquid chromatography tandem mass spectrometry (HPLC-MS/MS) system was operated using a Milli-Q purification system (Millipore, Bedford, MA, United States) throughout the analytical procedures.

For metabolite analysis, *β*-glucuronidase (type IX-A from *Escherichia coli*) with a glucuronidase activity ranging from 1,000,000 to 5,000,000 units/g protein (tested glucuronidase activity = 2,354,185 units/g protein, re-tested on 04/02/2020), and sulfatase (type H-1 from *Helix pomatia*) with a sulfatase activity of ≥10,000 units/g solid (containing sulfatase activity = 16,134 units/g solid and *β*-glucuronidase activity = 353,820 units/g solid, analysis date 25/05/2021) were obtained from Sigma-Aldrich (St. Louis, MO, United States). Sodium hydrogen phosphate, sodium dihydrogen phosphate, glacial acetic acid and sodium acetate were obtained from Sigma-Aldrich (St. Louis, MO, United States).

### 2.2 Study medication

The content of andrographolide in *A. paniculata* aqueous extract was determined by HPLC-DAD assay. The extract contained andrographolide not less than 4% w/w of dried powder extract. The assessment of quality control of herbal extract, including the limits of microbial contamination, heavy metals, and pesticides, were tested according to the THP 2021 guidelines (Department of Medical Sciences, 2021). *Andrographis paniculata* aqueous extract was formulated in capsules containing not less than 20 mg/capsule of andrographolide, and was manufactured by Panaosod Co., Ltd. (Thailand) under Good Manufacturing Practice (GMP) standards (Lot number 119010921). The quality control of *A. paniculata* capsules was consequently investigated as described by the THP 2021. This newly developed aqueous extract capsule was registered as a study medication for use in clinical trial and was approved by the Thai Food and Drug Administration (Thai FDA, Ministry of Public Health).

### 2.3 Determination of active diterpenoids in *Andrographis paniculata* aqueous extract capsules using high performance liquid chromatography photodiode array detection (HPLC-DAD)

*Andrographis paniculata* aqueous extract capsules were analyzed for the levels of the four major active diterpenoids using the previously described HPLC-DAD method (Pholphana et al., 2013). Briefly, 50 mg of *A. paniculata* aqueous extract in capsule (20 capsules) was accurately weighed in a 100 mL volumetric flask and extracted with methanol in an ultrasonic bath (Elma, Germany) for 30 min. The extracted solution was filtered through a 0.2 µm PVDF membrane (Chrom Tech, MN, United States). The four major active diterpenoids were simultaneously analyzed by HPLC-DAD (Agilent Technologies, Waldbronn, Germany) on a reverse phase column (Zorbax SB-C18; 4.6 × 75 mm, 3.5 μm) (Agilent Technologies, CA, United States), using 28% acetonitrile in water as the mobile phase delivered at a flow rate of 1.2 mL/min. The diode array detector was set at 205 nm. The contents of andrographolide; 14-deoxy-11, 12-didehydroandrographolide; neoandrographolide; and 14-deoxyandrographolide in *A. paniculata* aqueous extract capsule are presented in Table 1. The HPLC chromatograms of the four standard diterpenoids and *A. paniculata* aqueous extract are shown in Supplementary Figure S1.

**TABLE 1:** Four active diterpenoids contents in *Andrographis paniculata* aqueous extract capsules.

Compounds	Four active diterpenoids contents in *A. paniculata* aqueous extract capsules	
	Active diterpenoid contents mean ± SD (mg/g, n = 3)	Active diterpenoid contents mean ± SD (mg/capsule, n = 3)
1) andrographolide	37.64 ± 1.01	20.26 ± 0.54
2)14-deoxy-11, 12-didehydroandrographolide	12.30 ± 0.43	6.62 ± 0.23
3) neoandrographolide	9.81 ± 0.13	5.28 ± 0.07
4) 14-deoxyandrographolide	5.61 ± 0.17	3.02 ± 0.09
Average weight of total powder in capsule (mean ± SD, n = 20)	538.23 ± 21.34 (mg/capsule)	

### 2.4 Pharmacokinetic study

#### 2.4.1 Ethics statement

This study protocol was approved by the Ethics Review Committee for Research Involving Human Subjects at Chulabhorn Research Institute (IRB number: 062/2563, with the approval date of 28th August 2020) and was registered on Thaiclinicaltrials.org, https://www.thaiclinicaltrials.org/show/TCTR20210201005 (first registration date: 1st February 2021, TCTR20210201005) following the WHO International Clinical Trials Registry Platform (WHO-ICTRP). The clinical activities of this study were conducted at International Bio Service (IBS), Bangkok, Thailand, under the standards of Good Laboratory Practice (GLP) as well as the International Conference on Harmonization - Good Clinical Practice (ICH-GCP) and in accordance with the Declaration of Helsinki. The purposes and details of the study were clearly explained and all participants provided written informed consent prior to the study commencing.

#### 2.4.2 Study participants

Twenty-four healthy Thai participants (12 males and 12 females) aged between 18 and 55 years were enrolled in this study. Eligible participants were evaluated for their participation based on medical histories, physical examination, and clinical laboratory screening. The participants with BMI between 18.0 and 30.0 kg/m^2^, who had tested negative for coronavirus disease 2019 (COVID-19) by RT-PCR test, were recruited. Study participants with a history of excessive smoking (>10 cigarettes/day), or who had a history of alcoholism or of moderate drinking (>3 drinks/day) were excluded. Participants who were pregnant, breastfeeding, or planning to become pregnant during the study period were also excluded. Sample size was calculated according to a general guide for phase I clinical trials and the sample size estimation in clinical trials (Sakpal, 2010). The number of participants was calculated as *Z*_*1-α/2*_ = 1.96 and *γ =* 0.15 for 95% confidence.

#### 2.4.3 Study design

**FIGURE 1:**
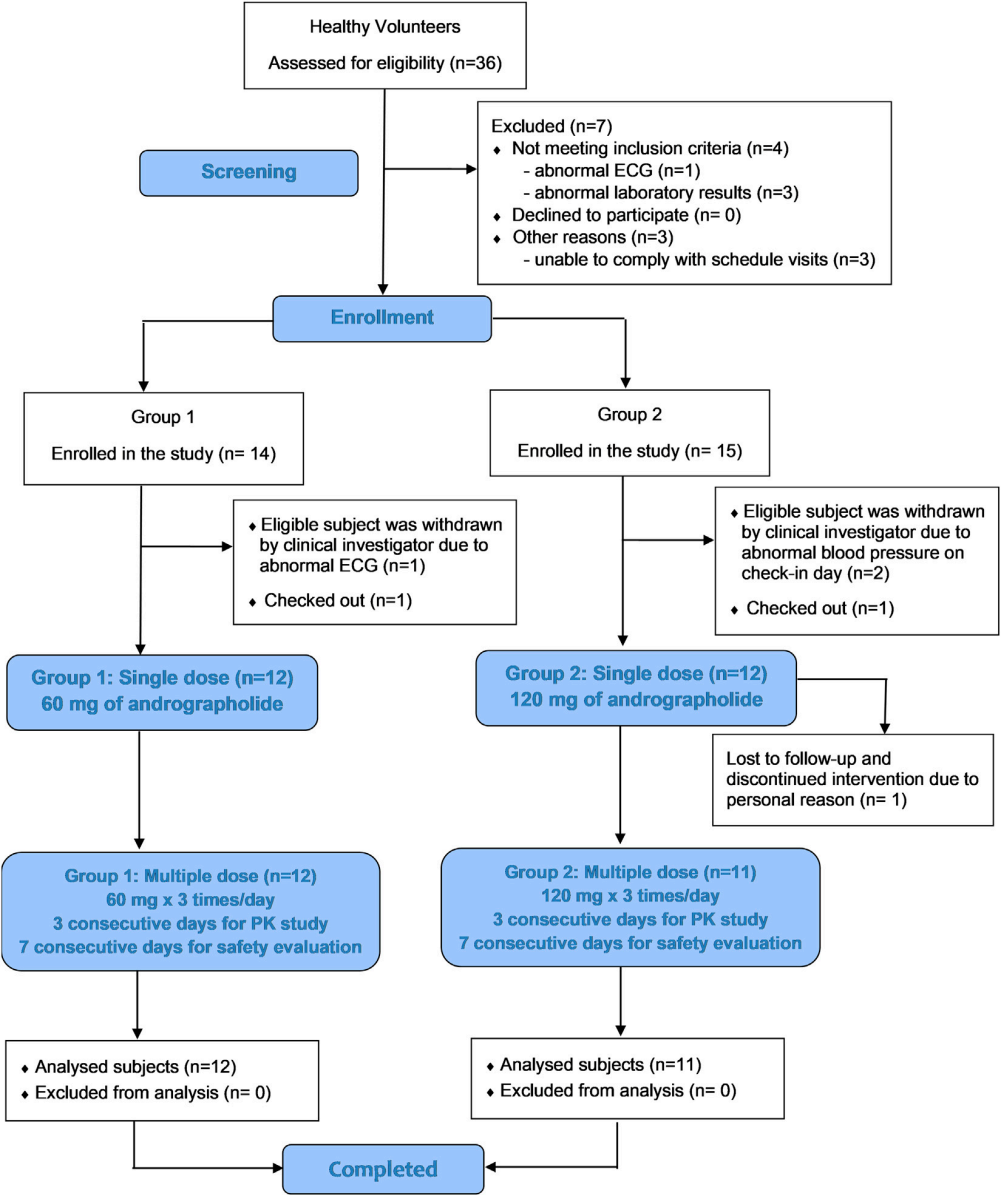
CONSORT schematic diagram of the study design.

An opened-label, two-dosage, single- and multiple-oral dosing, single center, phase I safety and pharmacokinetic study was conducted in healthy participants while they were under a fasting condition. As shown in Figure 1, twenty-four participants were recruited and randomized into two groups to receive *A. paniculata* aqueous extract capsules at equivalent doses of andrographolide of either 60 mg as single dose and 180 mg/day as multiple doses (group 1), or 120 mg as single dose and 360 mg/day as multiple doses (group 2). Two weeks prior to the beginning of the clinical study, the volunteers were advised to abstain from other dietary supplements, herbal products, or any concomitant medicines. During the period of the study, no other medications were allowed. All participants fasted overnight for at least 8 h before drug administration. The study conducted a single-dose investigation on day 1 by oral administering *A. paniculata* extract capsules once in both groups. Twelve participants in group 1 received 3 capsules orally (equivalent to 60 mg of andrographolide) with 240 mL of water, while another twelve participants in group 2 received 6 capsules (equivalent to 120 mg of andrographolide). This was followed by serial blood sampling for up to 24 h after dosing. For blood collection, 6 mL of venous blood in EDTA tube was collected via a forearm vein catheter at each time point including pre-dose (0 h), and post-dose after oral administration (T 0.167, 0.333, 0.5, 0.75, 1, 1.5, 2, 4, 6, 8, 10, 12 and 24 h). Subsequently, observation of elimination continued until 48 h post-dose to ensure complete excretion of the major active diterpenoids through urine. Urine samples were collected at day 0 (the day prior to dosing) and day 1–2 (T 0–4, 4–8, 8–12,12–24, 24–32, 32–40, and 40–48 h after dosing). The study then proceeded to investigate multiple doses in both groups by administering continuous oral doses from day 3 until the morning of day 5. (dosing at T48, 56, 64, 72, 80, 88, and 96 h). In group 1, three capsules were administered orally before meals, three times a day (3 × 3 capsules at 8-h intervals, calculated as 180 mg/day of andrographolide); whereas in group 2, six capsules were administered daily, three times a day (6 × 3 capsules at 8-h intervals, calculated as 360 mg/day of andrographolide). For serial blood sampling of multiple doses, 6 mL of blood samples were collected starting in the morning of day 5 (sampling timepoints T 96, 96.167, 96.333, 96.5, 96.75, 97, 97.5, 98, 100, 102, and 104 h post dose).

For safety evaluation during the study, the participants administered *A. paniculata* extract for a duration of 7 consecutive days. They arrived at the study site 8 h prior to the first dosing and were continuously monitored until 24 h after the initial dosing. They remained at the study site until day 5 of the study in order to observe any adverse events (AEs). On day 5, blood samples were collected for safety evaluation. For observation of any AEs occurred during this period, the participants were closely monitored and provided with standard treatment care, along with a comprehensive explanation of the AEs. To complete the 7 consecutive days of safety profiles, the participants received *A. paniculata* aqueous extract capsules for self-medication at home on day 6–9. On day 10, all participants returned to the study site for a follow-up visit and blood collection for safety evaluation. However, for those participants whose blood chemistry parameters were not returned to their individual baseline, they continued to be followed up for the next 7 days until they completely returned to their normal healthy baseline. The tolerability was assessed based on adverse events, vital signs monitoring, and blood chemistry testing, which continued to be followed up until the end of the study.

#### 2.4.4 Sample preparation

All collected blood samples were centrifuged at 4,000 rpm, 4^ο^C, for 5 min. Plasma was collected and stored in cryotubes at −70^ο^C until analysis. Urine was kept as 10 mL aliquot samples at each time point and stored at −70^ο^C until analysis. Extraction was performed by protein precipitation method as previously reported (Songvut et al., 2023). Briefly, 50 µL of either a plasma or urine sample was mixed with 200 µL of methanol containing an internal standard (IS) of digoxin (50 ng/mL). Pretreated samples were vortexed for 10 min and were then centrifuged at 12,000 rpm, 4°C, for 10 min. The supernatants were filtered through a 0.2 µm PVDF membrane and were subsequently transferred to vials for HPLC-MS/MS analysis.

Hydrolysis of the glucuronidated and sulfated conjugates was conducted by enzymatic incubation as our previous published method (Songvut et al., 2023). To analyze the metabolic conjugations, 50 μL of either a plasma or urine sample was pre-incubated at 37^ο^C with 50 µL of either of *β*-glucuronidase in sodium phosphate buffer (pH 6.8) for 30 min, or sulfatase in sodium acetate buffer (pH 5) for 2 h. IS (50 ng/mL) was then added along with 150 μL of methanol to precipitate protein. The extracted samples were then vortexed and centrifuged at 12,000 rpm, 4^ο^C, for 10 min. The supernatants were collected by filtering through a 0.2-µm PVDF membrane before HPLC-MS/MS injection.

#### 2.4.5 Quantitative determination by HPLC-MS/MS analysis

Analysis for the concentrations of targeted compounds in plasma and urine samples were carried out using HPLC-MS/MS at the Laboratory of Pharmacology, Chulabhorn Research Institute, Bangkok, Thailand. The quantitative determination was performed according to a previously validated method (Songvut et al., 2023).

The LC system consisted of an LC-40D XR pump unit (Shimadzu, Kyoto, Japan), SIL-40C XR autosampler and mass spectrometry was operated by LCMS-8060NX (Shimadzu, Kyoto, Japan) coupled with a triple quadrupole mass spectrometer and equipped with electrospray ionization (ESI) source. MS analysis was performed in negative ESI mode. The quantification was obtained in multiple-reaction monitoring (MRM) transition. Chromatographic separation was performed on a VertiSep AQS C18 (100 × 3.0 mm, 3 μm) (Vertical Chromatography, Thailand), protected by a vertical guard. Column oven temperature was maintained at 40°C during the analytical procedure. The extracted samples, 10 μL, were injected and eluted by gradient mobile phase system consisting of A) milli-Q and B) acetonitrile at a flow rate of 0.5 mL/min. The gradient was run as 40% B for 1.5 min, increased up to 100% B during 1.5–3.0 min, and maintained at 100% B over 3.0–4.5 min, and was then returned to the initial condition at 40% over 4.6–6.0 min. The m/z transitions (precursor/product ion) of 1) andrographolide, 2)14-deoxy-11, 12-didehydroandrographolide, 3) neoandrographolide, 4) 14-deoxyandrographolide, and digoxin (IS) were at 349.1/287.2, 331.1/239.2, 479.2/161.0, 333.1/285.2, and 779.3/649.2, respectively (Supplementary Figures S2 and S3). The instrument operations and the data acquisition were performed using Lab Solution software (Shimadzu, Kyoto, Japan).

#### 2.4.6 Data processing and pharmacokinetic analysis

All data on pharmacokinetics after oral administration were processed using Summit Research Services PK Solutions, software version 2.0. Non-compartmental analysis was used for determining all related pharmacokinetic parameters. Plasma concentration-time profiles in single and multiple doses were generated using GraphPad Prism 9.3.0 (GraphPad Software, United States). The maximum plasma concentration (C_max_) of each target compound and the time to reach maximum plasma concentration (T_max_) were directly observed and taken from the graphs. The area under the curve from time zero to the last quantifiable time point (AUC_0-t_) was calculated by linear trapezoidal rule, while the (AUC_0-inf_) was extrapolated to time infinity by using the formula AUC_(0-inf)_ = AUC_(0-t)_ +(C_last_/k_el_), where k_el_ is the elimination rate constant estimated by log-linear least square regression of the terminal phase, and C_last_ is the detectable concentration at the last blood sampling time point. The apparent volume of distribution (Vd/F) was determined according to the equation: Vd/F = Dose/(AUC x k_el_), where F is oral bioavailability. The apparent total clearance after oral administration (Cl/F) was calculated based on the equation: Cl/F = Dose/AUC. The elimination half-life (t_1/2_) was determined by t_1/2_ = ιn_2_/k_el_, when using ιn_2_ = 0.693.

Plasma and urine concentrations of the compounds 1) andrographolide, 2)14-deoxy-11, 12-didehydroandrographolide, 3) neoandrographolide, and 4) 14-deoxyandrographolide, in terms of glucuronidated and sulfated conjugates were determined according to the total concentrations of their unconjugated and conjugated forms. The corresponding free forms were also indicated prior to a hydrolysis reaction and the levels of conjugated metabolites were subsequently calculated.

### 2.5 Safety and tolerability evaluation

The assessment of safety and tolerability was carried out by monitoring adverse events (AEs), evaluating vital signs (blood pressure and heart rate), and blood chemistry testing. Adverse events were assessed based on the causality (relationship to the study medication) and categorized in terms of definitely related, possibly related, probably related, unlikely related, or unrelated. Then, the severity of each AE was evaluated and classified as mild, moderate, or severe. For blood chemistry testing, blood samples were obtained at the screening visit (day 0), on the fifth day following oral administration, and post-dose at the end of the study.

### 2.6 Statistical analysis

The primary outcome of this clinical study was to determine pharmacokinetic profiles and its parameters of the 4 mainly observed compounds (andrographolide; 14-deoxy-11, 12-didehydroandrographolide; neoandrographolide; and 14-deoxyandrographolide), and of their metabolites associated with the metabolic pathways of glucuronidation and sulfation. The secondary outcome was to evaluate the safety profile of the two different high doses of *A. paniculata* aqueous extract capsule orally administered at 60 or 120 mg, three times a day (equivalent to 180 or 360 mg/day of andrographolide) for 7 consecutive days.

Statistical analysis was performed using IBM SPSS version 22.0 (IBM SPSS Statistics, NY, United States) and the graphical charts were made using GraphPad Prism 9.3.0 (GraphPad Software). All data were tested for distribution using the Shapiro–Wilk test and then the histograms of the distribution data were considered. Continuous data with normal distribution were expressed as the mean ± standard deviation (SD), whereas T_max_ and t_1/2_ were expressed as the median (IQR). To compare the differences in PK parameters between 2 related single and multiple oral administrations, the data were analyzed by using a paired *t*-test or Wilcoxson Signed Rank Test, where appropriate. Student’s t-test or Mann-Whitney U test were also used when comparing between two doses (180 and 360 mg/day of andrographolide). All differences were considered statistically significant at *p*-values less than 0.05.

## 3 Results

### 3.1 Participant demographics and characteristics

Participant demographics and baseline characteristics are shown in Table 2. Thirty-six healthy participants underwent screening during the screening visit. Of these, twenty-nine participants were enrolled in the study, with group 1 consisting of 14 participants and group 2 consisting of 15 participants, as indicated in Figure 1 CONSORT Diagram. The participants ranged in age from 18 to 50 years. Among the enrolled participants, twelve subjects of group 1, including 6 men (50%) and 6 women (50%) with a mean age of 33.3 ± 11.1 years and a mean BMI of 22.18 ± 2.49 kg/m^2^, met the inclusion and exclusion criteria. In group 2, one participant (n = 1) discontinued due to personal reason and was lost to follow up. Thus, there were eleven participants who completed the study of group 2. Data from these participants was processed in terms of per-protocol analysis.

**TABLE 2:** Demographic characteristics of the study participants.

Demographic data		Group 1 (n = 12)		Group 2 (n = 11)	
		Single dose andrographolide 60 mg	Multiple dose andrographolide 60 mg × 3 times/day	Single dose andrographolide 120 mg	Multiple dose andrographolide 120 mg × 3 times/day
Gender, % (n)	Male	50% (n = 6)	50% (n = 6)	45.5% (n = 5)	45.5% (n = 5)
	Female	50% (n = 6)	50% (n = 6)	54.5% (n = 6)	54.5% (n = 6)
Age (year)		33.3 ± 11.1	33.3 ± 11.1	34.0 ± 11.3	34.0 ± 11.3
Body mass index (kg/m^2^)		22.18 ± 2.49	22.46 ± 2.47	22.66 ± 1.64	22.87 ± 1.62
Systolic blood pressure (mmHg)		119 ± 8	119 ± 7	110 ± 11	112 ± 11
Diastolic blood pressure (mmHg)		76 ± 6	77 ± 7	70 ± 8	73 ± 8
Body temperature (°C)		36.7 ± 0.3	36.8 ± 0.3	36.5 ± 0.3	36.5 ± 0.4
Respiratory rate (breaths/min)		20 ± 1	20 ± 1	19 ± 1	20 ± 1
Pulse rate (breaths/min)		80 ± 12	80 ± 8	70 ± 11	76 ± 11

Data are expressed as mean ± SD, (n = 11–12, one eligible participant was withdrawn by the clinical investigator due to personal reason).

Decimal numbers were reported according to laboratory standard of Clinical Research Center, Mahidol University.

### 3.2 Pharmacokinetic results

**FIGURE 2:**
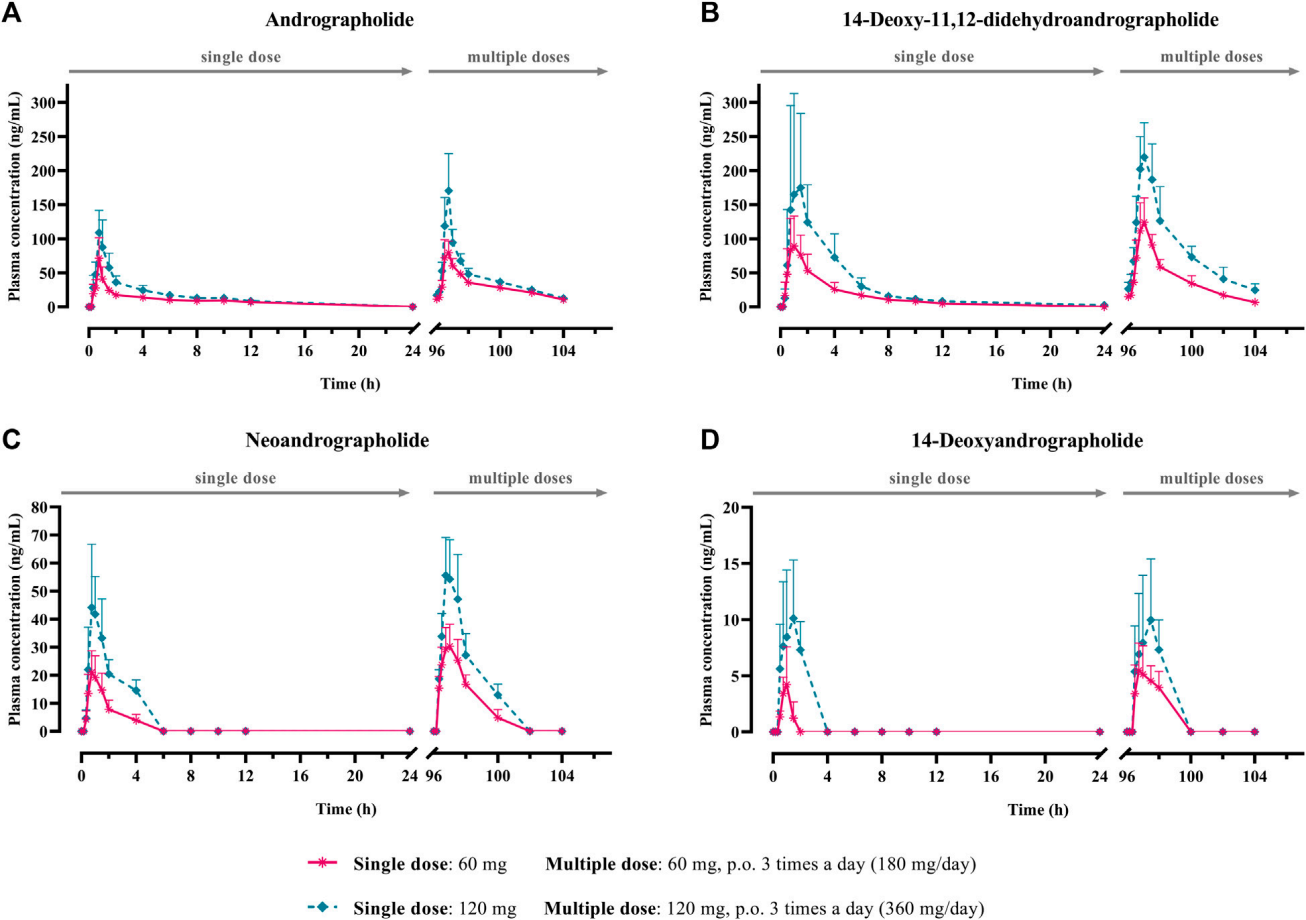
Mean plasma concentration-time profiles of parent diterpenoids; **(A)** andrographolide; **(B)** 14-deoxy-11, 12-didehydroandrographolide; **(C)** neoandrographolide; and **(D)** 14-deoxyandrographolide, after single (60 or 120 mg of andrographolide) and multiple (60 mg × 3 times or 120 mg × 3 times/day of andrographolide) oral administration of *Andrographis paniculata* aqueous extract capsules in healthy participants. Data are presented as the mean ± SD (n = 11–12).

**FIGURE 3:**
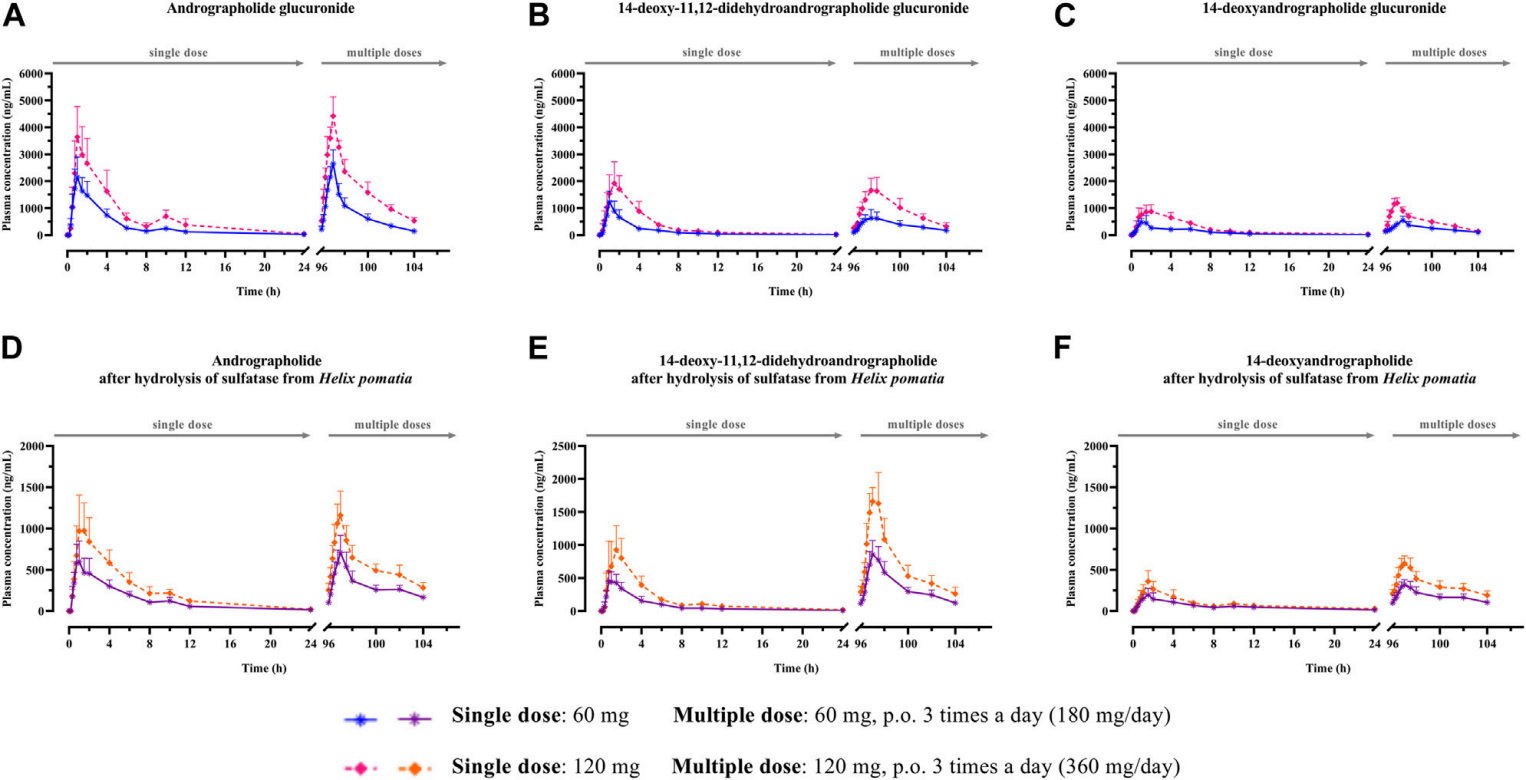
**(A–F)** Mean plasma concentration-time profiles of conjugated metabolites after single and multiple oral administration of *Andrographis paniculata* aqueous extract capsules in healthy participants. Data are presented as the mean ± SD (n = 11–12).

The mean plasma concentration-time profiles of the parent compounds and their metabolites after oral administration of *A. paniculata* aqueous extract capsule are presented in Figures 2, 3. All related pharmacokinetic parameters are shown in Tables 3–6.

**TABLE 3:** Summary of pharmacokinetic parameters of andrographolide and the conjugated metabolites following single and multiple oral administrations of *A. paniculata* aqueous extract capsules.

Single dose						
PK parameters	Andrographolide		Conjugated glucuronide^+^		Conjugated metabolites after hydrolysis with sulfatase^++^	
(T _0–24__ __h_)	60 mg (n = 12)	120 mg (n = 11)	60 mg (n = 12)	120 mg (n = 11)	60 mg (n = 12)	120 mg (n = 11)
Cmax (ng/mL)^a^	72.1±28.7	112±33.4*	2,481±592	3,843±1,050	637±253	1,036±357
Tmax (h)^b^	0.80 [0.00]	0.80 [0.00]	1.0[0.13]	1.0[0.00]	1.0[0.18]	1.5[0.63]
AUC_(0–24h)_ (ng-h/mL)^a^	210±25.4	345±63.5*	8,627±1,378	16,351±4,803	3,024±563	5,278±1,273
AUC_(0-inf)_ (ng-h/mL)^a^	210±25.4	345±63.5*	8,824±1,393	16,676±4,752	3,244±689	5,470±1,273
MRT (h)^a^	6.0±0.45	5.3±0.63	5.2±1.0	5.9±0.84	7.7±2.9	6.8±2.2
Vd/F (L/Kg)^a^	9.0±1.66	10.8±1.6	0.87±0.36	0.88±0.47	3.4±1.2	2.7±1.5
Cl/F (L/h/Kg)^a^	4.9±0.84	6.0±0.91	0.12±0.02	0.13±0.04	0.32±0.08	0.4±0.09
Half life (h)^b^	1.3[0.03]	1.2[0.05]	5.0[0.82]	4.1[1.6]	7.5[4.5]	4.2[3.0]
Urine (T _0–48__ __h_)						
Cumulative urine (μg)^a^	372±159	1,193±553	21,818±9,307	36,268±11,041	20,068.99±7,139.25	36,414.19±13,772.47

Multiple doses						
PK parameters	Andrographolide		Conjugated glucuronide^+^		Conjugated metabolites after hydrolysis with sulfatase ^++^	
(T _0–8_ _h_)	60 mg (n = 12)	120 mg (n = 11)	60 mg (n = 12)	120 mg (n = 11)	60 mg (n = 12)	120 mg (n = 11)
Cmax (ng/mL)^a^	85.9±19.0	172±54.4	2,783±354	4,483±635*	672±200	1,119.7±282*
Tmax (h)^b^	0.65[0.30]	0.80[0.00]	1.0[0.00]	1.0[0.00]	1.0[0.00]	1.0[0.10]
AUC_(0–8h)_ (ng-h/mL)^a^	195±24.0	345±48.8	6,338±1,004	14,010±1,707*	2,298±485	4,106±780*
MRT (h)^a^	4.3±0.99	3.4±0.45	4.0±3.3	3.9±0.60	6.8±3.4	6.3±2.3
Vd/F (L/Kg)^a^	18.0±8.0	14.7±3.7	0.61±0.76	0.47±0.17	1.8±0.91	2.0±0.56
Cl/F (L/h/Kg)^a^	4.5±0.86	5.5±1.1	0.15±0.03	0.13±0.02	0.33±0.08	0.38±0.10
Half life (h)^b^	2.3[1.0]	1.7[0.53]	1.5[1.6]	2.2[0.64]	3.0[2.1]	3.2[1.2]

Abbreviations: Cmax, maximum plasma concentration; Tmax, time to reach maximum concentration; AUC, area under the plasma concentration–time curve; MRT, mean residence time; Vd/F, the apparent volume of distribution; Cl/F, the apparent clearance; Half life, elimination half-life.

^a^ Data are expressed as mean ± SD, (n = 11–12).

^b^ Data is expressed as median [IQR].

^+^ Pharmacokinetic parameters of conjugated glucuronide metabolites after deconjugation using the β-glucuronidase enzyme type IX-A from *Escherichia coli* (containing glucuronidase activity).

^++^ Pharmacokinetic parameters of total conjugated sulfate and glucuronide metabolites, obtained from the total amount of the quantifiable compound after deconjugation using sulfatase enzyme type H-1 from *Helix pomatia* (containing sulfatase and glucuronidase activities), that has been subtracted by the parent free form of each detected compound.

Values less than 1: 2 decimals; values between 1 to 100: 1 decimal; values more than 100: no decimal

**TABLE 4:** Summary of pharmacokinetic parameters of 14-deoxy-11, 12-didehydroandrographolide and the conjugated metabolites following single and multiple oral administrations of *A. paniculata* aqueous extract capsules.

Single dose						
PK parameters	14-Deoxy-11,12-didehydroandrographolide		Conjugated glucuronide^+^		Conjugated metabolites after hydrolysis with sulfatase^++^	
(T _0–24__ __h_)	60 mg (n = 12)	120 mg (n = 11)	60 mg (n = 12)	120 mg (n = 11)	60 mg (n = 12)	120 mg (n = 11)
Cmax (ng/mL)^a^	98.6±40.3	211±138*	1,314±329	2,304±678	438±130	803±279
Tmax (h)^b^	1.0[0.33]	1.5[0.25]	1.0[0.13]	1.5[0.25]	0.90[0.70]	1.5[0.00]
AUC_(0–24h)_ (ng-h/mL)^a^	328±102	674±274*	3,507±809	8,174±1,585	1,632±330	3,449±888
AUC_(0-inf)_ (ng-h/mL)^a^	328±102	674±274*	3,711±866	8,529±1,636	1,793±401	3,607±873
MRT (h)^a^	4.3±0.44	4.1±0.65	6.2±1.3	6.0±1.6	8.5±5.0	6.7±1.4
Vd/F (L/Kg)^a^	2.1±0.60	2.0±0.69	1.2±0.50	0.93±0.57	2.7±1.6	1.8±0.7
Cl/F (L/h/Kg)^a^	1.1±0.27	1.1±0.36	0.09±0.02	0.08±0.01	0.19±0.04	0.19±0.03
Half life (h)^b^	1.4[0.07]	1.2[0.06]	9.7[4.7]	6.2[5.5]	8.0[3.4]	6.0[2.5]
Urine (T _0–48__ __h_)						
Cumulative urine (μg)^a^	70.6±27.6	159±56.8	11,215±3,710	20,446±6,091	7,819±2679	15,414±4,804

Multiple doses						
PK parameters	14-Deoxy-11,12-didehydroandrographolide		Conjugated glucuronide^+^		Conjugated metabolites after hydrolysis with sulfatase^++^	
(T _0–8_ _h_)	60 mg (n = 12)	120 mg (n = 11)	60 mg (n = 12)	120 mg (n = 11)	60 mg (n = 12)	120 mg (n = 11)
Cmax (ng/mL)^a^	137±29.3	237±41.4	769±272	1,743±479*	807±190	1,581±322*
Tmax (h)^b^	1.0[0.20]	1.0[0.33]	1.3[0.63]	1.5[0.50]	1.0[0.50]	1.0[0.50]
AUC_(0–8h)_ (ng-h/mL)^a^	329±55.5	693±165	3,102±954	7,549±1,995*	2,676±594	5,0240±1,305*
MRT (h)^a^	3.0±0.61	4.5±1.2	6.3±4.1	4.4±1.1	4.4±1.1	5.4±3.2
Vd/F (L/Kg)^a^	2.6±1.8	4.4±2.0	0.45±0.26	0.25±0.08	0.39±0.20	0.52±0.20
Cl/F (L/h/Kg)^a^	0.98±0.23	0.85±0.14	0.09±0.03	0.08±0.02	0.11±0.02	0.11±0.03
Half life (h)^b^	1.6[0.80]	3.0[1.5]	2.8[1.9]	1.8[1.4]	2.4[1.8]	2.6[2.3]

Abbreviations: Cmax, maximum plasma concentration; Tmax, time to reach maximum concentration; AUC, area under the plasma concentration–time curve; MRT, mean residence time; Vd/F, the apparent volume of distribution; Cl/F, the apparent clearance; Half life, elimination half-life.

a Data are expressed as mean ± SD, (n = 11–12).

^b^ Data is expressed as median [IQR].

^+^ Pharmacokinetic parameters of conjugated glucuronide metabolites after deconjugation using the β-glucuronidase enzyme type IX-A from *Escherichia coli* (containing glucuronidase activity).

^++^ Pharmacokinetic parameters of total conjugated sulfate and glucuronide metabolites, obtained from the total amount of the quantifiable compound after deconjugation using sulfatase enzyme type H-1 from *Helix pomatia* (containing sulfatase and glucuronidase activities), that has been subtracted by the parent free form of each detected compound.

Values less than 1: 2 decimals; values between 1 to 100: 1 decimal; values more than 100: no decimal

**TABLE 5:** Summary of pharmacokinetic parameters of neoandrographolide and the conjugated metabolites following single and multiple oral administrations of *A. paniculata* aqueous extract capsules.

Single dose (T _0–24 h_)						
	Neoandrographolide		Conjugated glucuronide^+^		Conjugated metabolites after hydrolysis with sulfatase^++^	
PK parameters	60 mg (n = 12)	120 mg (n = 11)	60 mg (n = 12)	120 mg (n = 11)	60 mg (n = 12)	120 mg (n = 11)
Cmax (ng/mL)^a^	24.4±5.6	50.4±19.2	N/A^#^	N/A^#^	N/A^#^	N/A^#^
Tmax (h)^b^	0.90[0.33]	0.80[0.20]	N/A^#^	N/A^#^	N/A^#^	N/A^#^
AUC_(0–24h)_ (ng-h/mL)^a^	41.0±4.9	104±22.6	N/A^#^	N/A^#^	N/A^#^	N/A^#^
AUC_(0-inf)_ (ng-h/mL)^a^	41.0±4.9	104±22.6	N/A^#^	N/A^#^	N/A^#^	N/A^#^
MRT (h)^a^	1.8±0.33	2.2±0.22	N/A^#^	N/A^#^	N/A^#^	N/A^#^
Vd/F (L/Kg)^a^	0.74±0.16	1.5±0.33	N/A^#^	N/A^#^	N/A^#^	N/A^#^
Cl/F (L/h/Kg)^a^	2.2±0.33	5.3±1.2	N/A^#^	N/A^#^	N/A^#^	N/A^#^
Half life (h)^b^	0.23[0.04]	0.19[0.01]	N/A^#^	N/A^#^	N/A^#^	N/A^#^
Urine (T _0–48 h_)						
Cumulative urine (μg)^a^	24.0±18.1	66.7±29.3	217±78.8	383±127	N/A^#^	N/A^#^

Multiple doses (T_0–8_ _h_)						
	Neoandrographolide		Conjugated glucuronide ^+^		Conjugated metabolites after hydrolysis with sulfatase ^++^	
PK parameters	60 mg (n = 12)	120 mg (n = 11)	60 mg (n = 12)	120 mg (n = 11)	60 mg (n = 12)	120 mg (n = 11)
Cmax (ng/mL)^a^	34.8±6.3	60.7±12.2	N/A^#^	N/A^#^	N/A^#^	N/A^#^
Tmax (h)^b^	1.0[0.33]	1.0[0.45]	N/A^#^	N/A^#^	N/A^#^	N/A^#^
AUC_(0–8h)_ (ng-h/mL)^a^	69.7±12.9	128±29.1	N/A^#^	N/A^#^	N/A^#^	N/A^#^
AUC_(0-inf)_ (ng-h/mL)^a^	69.7±12.9	128±29.1	N/A^#^	N/A^#^	N/A^#^	N/A^#^
MRT (h)^a^	1.7±0.22	1.9±0.18	N/A^#^	N/A^#^	N/A^#^	N/A^#^
Vd/F (L/Kg)^a^	3.4±0.78	1.2±0.29	N/A^#^	N/A^#^	N/A^#^	N/A^#^
Cl/F (L/h/Kg)^a^	3.9±0.88	4.3±0.95	N/A^#^	N/A^#^	N/A^#^	N/A^#^
Half life (h)^b^	0.60[0.00]	0.19[0.01]	N/A^#^	N/A^#^	N/A^#^	N/A^#^

Note: ^#^Due to most plasma concentrations of the conjugated forms of neoandrographolide being undetectable until the elimination, and others being below the quantifiable limit, PK parameters of its conjugated metabolites were not included in the table.

Abbreviations: Cmax, maximum plasma concentration; Tmax, time to reach maximum concentration; AUC, area under the plasma concentration–time curve; MRT, mean residence time; Vd/F, the apparent volume of distribution; Cl/F, the apparent clearance; Half life, elimination half-life.

^a^ Data are expressed as mean ± SD, (n = 11–12).

^b^ Data is expressed as median [IQR].

^+^ Pharmacokinetic parameters of conjugated glucuronide metabolites after deconjugation using the β-glucuronidase enzyme type IX-A from *Escherichia coli* (containing glucuronidase activity).

^++^ Pharmacokinetic parameters of total conjugated sulfate and glucuronide metabolites, obtained from the total amount of the quantifiable compound after deconjugation using sulfatase enzyme type H-1 from *Helix pomatia* (containing sulfatase and glucuronidase activities), that has been subtracted by the parent free form of each detected compound.

Values less than 1: 2 decimals; values between 1 to 100: 1 decimal; values more than 100: no decimal

**TABLE 6:** Summary of pharmacokinetic parameters of 14-deoxyandrographolide and the conjugated metabolites following single and multiple oral administrations of *A. paniculata* aqueous extract capsules.

Single dose						
PK parameters	14-Deoxyandrographolide		Conjugated glucuronide^+^		Conjugated metabolites after hydrolysis with sulfatase^++^	
(T _0–24__ __h_)	60 mg (n = 12)	120 mg (n = 11)	60 mg (n = 12)	120 mg (n = 11)	60 mg (n = 12)	120 mg (n = 11)
Cmax (ng/mL)^a^	4.2±3.4	11.6±5.4*	578±248	981±262	215±67.0	372±113
Tmax (h)^b^	1.0[0.33]	1.0[0.70]	1.0[0.00]	1.8[0.50]	1.5[0.50]	1.5[0.13]
AUC_(0–24h)_ (ng-h/mL)^a^	N/A^#^	20.5±8.8*	2,549±553	5,874±1,040	1,332±308	2,112±531
AUC_(0-inf)_ (ng-h/mL)^a^	N/A^#^	20.5±8.8*	2,713±610	6,372±1,187	1,618±518	2,678±774
MRT (h)^a^	N/A^#^	1.5±0.08	7.6±1.5	8.3±2.8	13.0±8.2	14.8±7.9
Vd/F (L/Kg)^a^	N/A^#^	5.5±2.9	0.61±0.22	0.61±0.26	1.2±0.69	1.7±0.72
Cl/F (L/h/Kg)^a^	N/A^#^	17.6±8.1	0.06±0.01	0.05±0.01	0.10±0.03	0.12±0.04
Half life (h)^b^	N/A^#^	0.21[0.01]	7.3[3.2]	7.6[7.1]	5.8[9.0]	10.1[7.3]
Urine (T _0–48_ _h_)						
Cumulative urine (μg)^a^	108±36.5	199±77.3	4,854±1,601	9,409±2,342	4,027±1,657	8,394±2,284

Multiple doses						
PK parameters	14-Deoxyandrographolide		Conjugated glucuronide ^+^		Conjugated metabolites after hydrolysis with sulfatase ^++^	
(T _0–8_ _h_)	60 mg (n = 12)	120 mg (n = 11)	60 mg (n = 12)	120 mg (n = 11)	60 mg (n = 12)	120 mg (n = 11)
Cmax (ng/mL)^a^	6.6±1.9	11.3±5.5	572±126	1,323±125*	333±70.1	596±79.9*
Tmax (h)^b^	1.0[0.32]	1.0[0.70]	1.5[0.00]	1.0[0.20]	1.0[0.33]	1.0[0.35]
AUC_(0–8h)_ (ng-h/mL)^a^	11.3±3.3	20.0±9.1	2,080±409	4,221±413*	1,480±318	2,597±498*
MRT (h)^a^	1.5±0.11	1.6±0.07	4.8±1.2	3.5±0.29	6.0±1.1	7.3±2.6
Vd/F (L/Kg)^a^	5.4±3.1	5.7±2.8	0.24±0.11	0.16±0.07	0.38±0.15	0.51±0.13
Cl/F (L/h/Kg)^a^	15.3±7.3	18.1±7.7	0.06±0.02	0.07±0.01	0.08±0.02	0.08±0.02
Half life (h)^b^	0.23[0.01]	0.21[0.02]	2.6[0.66]	1.6[0.73]	3.4[1.1]	4.2[3.0]

Note: ^#^Pharmacokinetic parameters were not reported due to most of the plasma concentration values being below the limit of quantification (LLOQ) and some being negligible.

Abbreviations: Cmax, maximum plasma concentration; Tmax, time to reach maximum concentration; AUC, area under the plasma concentration–time curve; MRT, mean residence time; Vd/F, the apparent volume of distribution; Cl/F, the apparent clearance; Half life, elimination half-life.

^a^ data are expressed as mean ± SD, (n = 11–12).

^b^ data is expressed as median [IQR].

^+^ Pharmacokinetic parameters of conjugated glucuronide metabolites after deconjugation using the β-glucuronidase enzyme type IX-A from *Escherichia coli* (containing glucuronidase activity).

^++^ Pharmacokinetic parameters of total conjugated sulfate and glucuronide metabolites, obtained from the total amount of the quantifiable compound after deconjugation using sulfatase enzyme type H-1 from *Helix pomatia* (containing sulfatase and glucuronidase activities), that has been subtracted by the parent free form of each detected compound.

Values less than 1: 2 decimals; values between 1 to 100: 1 decimal; values more than 100: no decimal

#### 3.2.1 Pharmacokinetic profiles of *Andrographis paniculata* aqueous extract capsule

##### 3.2.1.1 Absorption and biotransformation

The AUC values of the parent compounds, which indicate the degree of exposure in the body, were much lower than those of their respective conjugated metabolites (Tables 3–6; Figures 2, 3). The mean values for C_max_ of the parent diterpenoids showed lower values than those of their respective metabolites. Noticeably, T_max_ values of the parent diterpenoids ranged between 0.65 and 1.50 h after dosing and were consistent with the results obtain in previous pharmacokinetic studies indicating that the T_max_ of andrographolide was 1.36 h (Panossian et al., 2000).

Regarding the pharmacokinetic characteristics of their metabolites, the highest concentrations of the glucuronide and sulfate metabolites were detected at 0.90–1.8 h after dosing. The results indicated that the concentration of conjugated metabolites is higher than that of the parent diterpenoids. The AUCs of the metabolites were found to be approximately 10–100 times higher than those of the parent compounds. These data suggested that the major diterpenoids were conjugated as glucuronide and sulfate derivatives in the systemic blood circulation, with the exception of neoandrographolide, which was less detectable as conjugated metabolites in plasma.

##### 3.2.1.2 Excretion

**FIGURE 4:**
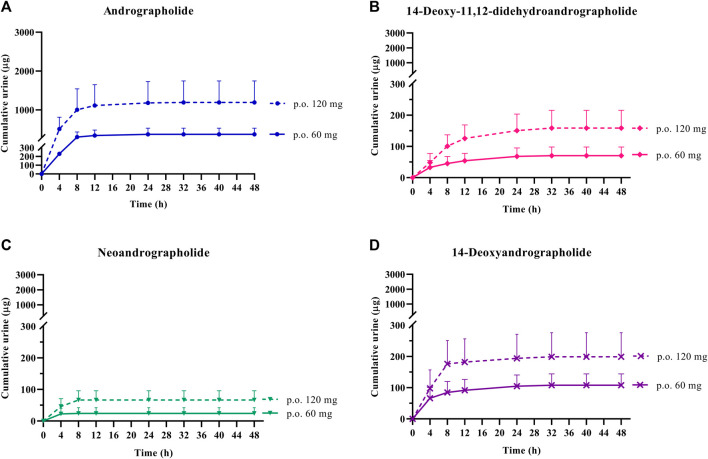
Cumulative urinary excretion of parent diterpenoids; **(A)** andrographolide; **(B)** 14-deoxy-11, 12-didehydroandrographolide; **(C)** neoandrographolide; and **(D)** 14-deoxyandrographolide, after single oral administration of *Andrographis paniculata* aqueous extract capsules (calculated as 60 or 120 mg of andrographolide) in healthy participants. Data are presented as the mean ± SD (n = 11–12).

**FIGURE 5:**
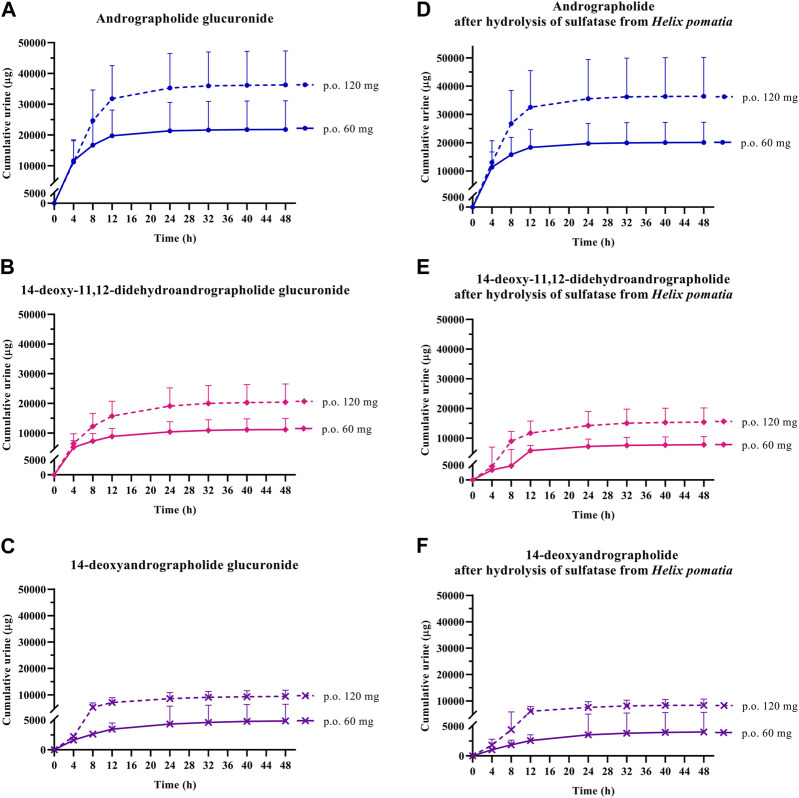
Cumulative urinary excretion of metabolites; **(A–C)** conjugated glucuronide and **(D–F)** conjugated metabolites after hydrolysis of sulfatase from *Helix pomatia*, following single oral administration of *Andrographis paniculata* aqueous extract capsules (calculated as 60 or 120 mg of andrographolide) in healthy participants. Data are presented as the mean ± SD (n = 11–12).

In the termination phase, all four bioactive compounds were mainly eliminated as both their unchanged (Figure 4 **)** and changed forms (Figure 5). The unchanged parent compounds were primarily excreted renally. These compounds were extensively biotransformed by the hepatobiliary system to become the glucuronide and sulfate derivatives, which were finally eliminated into the urine until 48 h after oral dosing. The highest amounts of the parent compounds in the urine were detected within 4–8 h post-dose, whereas the conjugated forms were intensively eliminated through the kidney within 8–12 h post-dose. Based on the pharmacokinetic parameters of the different doses, there were no significant changes observed in elimination half-life (t_1/2_) for all four parent compounds and their conjugated metabolites, even when the extract was administered at a higher dose. Likewise, the apparent clearance (Cl/F) did not show significant differences when comparing these two doses by non-compartmental analysis.

#### 3.2.2 Dosage regimen comparison

The mean C_max_ and AUC_(0–24h)_ after single oral administration of *A. paniculata* aqueous extract capsule equivalent to 60 mg of andrographolide were compared to those obtained after administration of 120 mg of andrographolide. Multiple oral administration of 60-mg doses three times a day (calculated as 180 mg/day of andrographolide) and 120-mg doses three times a day (calculated as 360 mg/day of andrographolide) for 3 consecutive days, were also compared for the mean C_max_ and AUC_(0-∞)_ of the repeated dose at steady state.

The results for the four parent diterpenoids showed that the AUC_(0–24h)_ following 120-mg single oral administration was approximately 2 times higher than that obtained from 60-mg single oral administration. Moreover, the AUC_(0-∞)_ for 360 mg/day orally administered as a repeated doses was approximately twofold greater than that obtained following administration of 180 mg/day as a multiple dose. Considering the dose proportionality of conjugated metabolites, those AUC(s) for the glucuronide and sulfate derivatives were increased proportionally when comparing values for 60 vs. 120 mg/day doses and 180 vs. 360 mg/day doses (as shown in Tables 3–6). The finding demonstrated that the parent compounds and their conjugated metabolites indicated dose-dependent plasma concentrations after doubling the administered doses. When considering urine excretion, doubling the oral dose of andrographolide also led to a nearly corresponding doubling of the amount excreted in urine.

### 3.3 Safety and tolerability

Aqueous extract capsules of *A. paniculata* orally administered for 7 days was found to be well tolerated in healthy participant throughout the study period. There were no serious incidents reported following repeated oral administration of either 180 or 360 mg/day of andrographolide (Table 7). The most commonly observed product-related adverse event (AE), was mild elevation in bicarbonate levels, which was reported in 11 out of 23 participants (n = 11/23; 47.83%). Other most commonly observed AEs were the increases of the following values: alanine aminotransferase or ALT levels (n = 7/23; 30.43%), total cholesterol levels (n = 7/23; 30.43%), aspartate aminotransferase or AST levels (n = 6/23; 26.09%), and triglyceride levels (n = 6/23; 26.09%). Some possible drug-related adverse events, affecting 1 out of 23 participants (n = 1/23; 4.35%), were reported in the group receiving a 360-mg oral dose, including mild decreases in creatinine, hematocrit or lymphocyte levels; or increases in neutrophil or platelet counts. Two participants experienced gastrointestinal disorders, including one with mild nausea, vomiting and mild diarrhea, after receiving a daily high dose of 360 mg/day of andrographolide, and the other experienced with mild loose stool after receiving 180 mg/day of andrographolide. In addition, one participant was reported mild dizziness along with postural hypotension. It was observed that elevated AST and ALT levels in participants could be returned to their normal baseline levels within 2–3 weeks and other clinical blood chemistry abnormalities were also recovered to their initial baseline levels within 1–2 weeks, even in the high-dose group (equivalent to 360 mg/day of andrographolide).

**TABLE 7:** Safety data and adverse events of *A. paniculata* aqueous extract capsules after multiple oral administration in different dosage regimens.

Adverse events	*A. paniculata* aqueous extract oral administration						
	180 mg/day (n = 12) n/12 (%) severity		360 mg/day (n = 11) n/11 (%) severity		Total (n = 23) n/23 (%) severity		Relation to medication
Gastrointestinal disorders							
Diarrhea	0/12		1/11 (9.09%)	mild	1/23 (4.35%)	mild	possibly
Loose stool	1/12 (8.33%)	mild	0/12		1/23 (4.35%)	mild	possibly
Nausea	0/12		1/11 (9.09%)	mild	1/23 (4.35%)	mild	possibly
Vomiting	0/12		1/11 (9.09%)	mild	1/23 (4.35%)	mild	possibly
Investigations							
ALT (SGPT) increased	4/12 (33.33%)	mild	3/11 (27.27%)	mild	7/23 (30.43%)	mild	probably
AST (SGOT) increased	4/12 (33.33%)	mild	2/11 (18.18%)	mild	6/23 (26.09%)	mild	probably
Bicarbonate increased	5/12 (41.67%)	mild	6/11 (54.55%)	mild	11/23 (47.83%)	mild	possibly
Chloride decreased	1/12 (8.33%)	mild	2/11 (18.18%)	mild	3/23 (13.04%)	mild	possibly
Creatinine decreased	0/12		1/11 (9.09%)	mild	1/23 (4.35%)	mild	possibly
HDL decreased	1/12 (8.33%)	mild	2/11 (18.18%)	mild	3/23 (13.04%)	mild	possibly
Hematocrit decreased	3/12 (25.00%)	mild	1/11 (9.09%)	mild	4/23 (17.39%)	mild	possibly
Hemoglobin decreased	3/12 (25.00%)	mild	2/11 (18.18%)	mild	5/23 (21.74%)	mild	possibly
Lymphocyte decreased	0/12		1/11 (9.09%)	mild	1/23 (4.35%)	mild	possibly
Neutrophil increased	0/12		1/11 (9.09%)	mild	1/23 (4.35%)	mild	possibly
Platelet count increased	0/12		1/11 (9.09%)	mild	1/23 (4.35%)	mild	possibly
Total cholesterol increased	5/12 (41.67%)	mild	2/11 (18.18%)	mild	7/23 (30.43%)	mild	possibly
Metabolism and nutrition disorders							
Albumin increased	2/12 (16.67%)	mild	0/12		2/23 (8.70%)	mild	possibly
Potassium decreased	1/12 (8.33%)	mild	2/11 (18.18%)	mild	3/23 (13.04%)	mild	possibly
Sodium decreased	0/12		1/11 (9.09%)	mild	1/23 (4.35%)	mild	possibly
Triglycerides increased	3/12 (25.00%)	mild	3/11 (27.27%)	mild	6/23 (26.09%)	mild	possibly
Nervous system disorders							
Dizziness	0/12		1/11 (9.09%)	mild	1/23 (4.35%)	mild	probably
Pregnancy, puerperium and perinatal conditions							
Exposure *in Utero*	0/12		1/11 (9.09%)	mild	1/23 (4.35%)	mild	unrelated
Vascular disorders							
Postural Hypotension	0/12		1/11 (9.09%)	mild	1/23 (4.35%)	mild	probably

AST, aspartate aminotransferase; ALT, alanine aminotransferase.

SGOT, glutamic-oxalacetic transaminase; SGPT, glutamic-pyruvic transaminase.

In order to evaluate the effects of *A. paniculata* aqueous extract on kidney and liver functions, a gender subgroup analysis was conducted as part of the clinical laboratory monitoring. Specifically, a comparison was made between pre-dose and post-dose measurements, as indicated in Table 8. Gender-specific differences were noted in the levels of total bilirubin, with significantly lower levels observed in males when compared to the predose. After the oral administration equivalent to 180 mg/day of andrographolide, both AST and ALT levels showed significant elevation in both male and female participants. This implies that there were no substantial differences in AST and ALT levels between males and females. However, in the group that received 360 mg/day of andrographolide, gender-specific differences became apparent. Females displayed a significant increase in ALT levels, while males did not exhibit significant differences in ALT levels at this higher dosage.

**TABLE 8:** Gender subgroup analysis of clinical laboratory monitoring of kidney and liver functions compared between pre-dose and post-dose of *A. paniculata* aqueous extract capsules.

Clinical laboratory parameters		References range	Andrographolide 180 mg/day		
			Baseline (male, n = 6) (Female, n = 6)	Day 5 (male, n = 6) (Female, n = 6)	Day 10 (male, n = 6) (Female, n = 6)
	Gender				
BUN (mg/dL)	Male	6–20	12±3	12±1	13±3
	Female	6–20	10±2	9±2	10±2
Creatinine (mg/dL)	Male	0.67–1.17	0.92±0.15	0.86±0.10	0.88±0.10
	Female	0.51–0.95	0.70±0.06	0.66±0.07	0.64±0.05
Total bilirubin (mg/dL)	Male	0.0–1.2	0.9±0.7	0.7±0.3	0.5±0.2 *
	Female	0.0–1.2	0.4±0.1	0.4±0.2	0.3±0.1
AST (U/L)	Male	0–40	20±3	27±11 *	27±13 *
	Female	0–32	24±11	23±9	40±32 *
ALT (U/L)	Male	0–41	23±13	33±20 *	39±29 *
	Female	0–33	16±5	17±11	38±33 *
Alkaline phosphatase (U/L)	Male	40–130	63±15	66±16	70±13
	Female	35–105	71±29	68±28	72±26
Albumin (g/dL)	Male	3.5–5.2	5±0	5±0	5±0
	Female	3.5–5.2	5±0	4±0 *	5±0

Clinical laboratory parameters		References Range	Andrographolide 360 mg/day		
			Baseline (Male, n = 5) (Female, n = 6)	Day 5 (Male, n = 5) (Female, n = 6)	Day 10 (Male, n = 5) (Female, n = 6)
	Gender				
BUN (mg/dL)	Male	6–20	12±2	10±0	12±2
	Female	6–20	11±3	10±2	11±2
Creatinine (mg/dL)	Male	0.67–1.17	0.94±0.04	0.86±0.07	0.89±0.12
	Female	0.51–0.95	0.69±0.09	0.60±0.09	0.67±0.10
Total bilirubin (mg/dL)	Male	0.0–1.2	0.6±0.2	0.6±0.2	0.5±0.2
	Female	0.0–1.2	0.4±0.2	0.4±0.2	0.4±0.1
AST (U/L)	Male	0–40	25±6	28±17	27±9
	Female	0–32	17±2	23±7	22±5
ALT (U/L)	Male	0–41	33±12	33±22	35±16
	Female	0–33	11±2	21±10	28±12 *
Alkaline phosphatase (U/L)	Male	40–130	74±13	70±12	74±12
	Female	35–105	49±20	51±20	53±21
Albumin (g/dL)	Male	3.5–5.2	5±0	5±0	5±0
	Female	3.5–5.2	5±0	4±0 *	5±0

Data are expressed as mean ± SD, (n = 11–12).

*A *p-*value of less than 0.05 was considered statistically significant.

Decimal numbers were reported according to laboratory standard of Clinical Research Center, Mahidol University.

AST, aspartate aminotransferase; ALT, alanine aminotransferase; BUN, blood urea nitrogen.

## 4 Discussion

Pharmacokinetics has been used to determine a drug’s systemic exposure after administration for achieving the desired therapeutic effects and clinical outcomes. Although the aqueous extract of *A. paniculata* has been traditionally used, there has been limited investigation into its pharmacokinetic profiles. This study presented the clinical pharmacokinetics of high doses of the aqueous extract of capsules *A. paniculata* focusing on the four major targeted diterpenoids (andrographolide, 14-deoxy-11, 12-didehydroandrographolide, neoandrographolide, and 14-deoxyandrographolide). Additionally, this research also investigated the presence of conjugated metabolites in plasma and their excretion in urine.

Following oral administration of *A. paniculata* aqueous extract, the pharmacokinetic profiles of comparative dosage showed that greater systemic exposure was observed in terms of conjugated metabolites that exhibited a slightly longer half-life (t_1/2_), when compared to their parent compounds. The finding indicated a slower elimination process for the metabolites. These characteristics strengthen the hypothesis of our previous study (Songvut et al., 2023) that the major parent diterpenoids are partly biotransformed by phase II metabolism into the conjugated glucuronide and sulfate metabolites. Furthermore, the evidences indicated glucuronidation and sulfation have been shown to occur in metabolic pathways of andrographolide (Coon and Ernst, 2004; Cui et al., 2005; Ye et al., 2011; Bera et al., 2014). A previous study suggested that the limited oral bioavailability of andrographolide may be attributed to its rapid biotransformation and efflux by P-glycoprotein (Ye et al., 2011). These conjugated forms have the potential to exhibit biological effects that may be associated with the efficient delivery of the active parent compounds to targeted organs. The importance of the formation of conjugated metabolites after oral administration of *A. paniculata* aqueous extract remains undetermined. Nonetheless, the presence of these glucuronide and sulfate conjugates is likely to be significant for future studies exploring the biological effects of these metabolites. This finding emphasizes the need for additional investigation into the pharmacological properties of the conjugated metabolites to justify their potential therapeutic benefits after oral administration of *A. paniculata*. Further investigation into the tissue distribution of these conjugated metabolites is necessary and should be undertaken using preclinical (*in vivo*) animal models.

For the termination phase, parent compounds and their conjugated metabolites of andrographolide, 14-deoxy-11, 12-didehydroandrographolide, and 14-deoxyandrographolide are excreted partly via urinary excretion. The previously available review reported that the metabolism and elimination pathways of active diterpenoids are associated with the hepatobiliary and renal systems (Songvut et al., 2022). The present study indicated that doubling the oral dose of andrographolide resulted in a proportional increase of nearly twice the amount excreted in the urine, suggesting a linear relationship between the dose and excretion of andrographolide. Dosage selection should take this association into account, especially when this plant is used in SARS-CoV-2 patients with hepatic or renal dysfunctions. Therefore, the appropriate dosage for use of *A. paniculata* extract in patients with hepatic or renal impairments should be further examined. Taking into account the remarkable presence of conjugated metabolites of active diterpenoids in plasma and urine, one notable exception was observed in the conjugation of neoandrographolide. The indirect method of enzyme hydrolysis reaction could enable the detection of a small amount of quantifiable conjugated neoandrographolide metabolites and some plasma concentration values were found to below the quantifiable limit. The presence of a sugar moiety in its chemical structure could potentially make it difficult for the compound to undergo conjugation at the position of interest, which could be a reason for the low levels of its conjugated metabolites (Songvut et al., 2023). However, it is still possible for neoandrographolide to undergo conjugation at other positions. Therefore, further study is essential to perform a direct analysis to investigate the biotransformation of this compound via the conjugation in phase II metabolic pathway.

Comparison of the two double doses (andrographolide 60 vs. 120 mg single dose and 180 vs. 360 mg/day multiple doses) indicated a twofold increase in the plasma levels of the major active diterpenoids and their conjugated metabolites following oral administration of a double dose of andrographolide. This finding could be further applied to determining and adjusting the appropriate these two dosage regimens of *A. paniculata* aqueous extract in patients. However, the study by Panossian et al. (2000) found that increasing the dose of *A. paniculata* extract by tenfold caused only a twofold increase in AUC_(0-∞)_ when using the one-compartment model. This finding suggested that *A. paniculata* extract may not exhibit dose proportionality at extremely different high dosages, possibly due to limitations in its absorption into the systemic circulation. For further in-depth study, it is suggested to include three or more doses to investigate a linear relationship for determining dose linearity of these compounds.

Taking into account the potential accumulation of active diterpenoids, since the half-life of each diterpenoid was less than 2 h and the dosing interval was 8 h, *A. paniculata* aqueous extract capsules were administered for a duration exceeding 5 half-lives, resulting in no observed accumulation. Furthermore, the results of this present study indicated that the AUC for multiple doses of each diterpenoid did not demonstrate a significant increase in comparison to the AUC for a single dose. Hence, the administration of repeated doses of *A. paniculata* aqueous extract, either 60 or 120 mg of andrographolide three times daily from day 3 to day 5, did not result in significant accumulation of the bioactive compounds. However, it maintained their plasma concentration, thereby ensuring the sustained therapeutic benefits of andrographolide.

In terms of safety and tolerability, the previous clinical studies of orally administered andrographolide of 60 mg/day for the treatment of URTIs have demonstrated that *A. paniculata* generally exhibited good safety and tolerance (Melchior et al., 2000; Gabrielian et al., 2002; Saxena et al., 2010). Our present study which administered significantly higher doses compared to the typical dose of traditional uses, found that 7 of 24 participants (n = 7/24; 29.17%) who received a daily dose of 180 mg/day or 360 mg/day of andrographolide experienced a mild increase in ALT compared to their baseline levels. This is consistent with a previous study indicating that patients with gastrointestinal problems who administered *A. paniculata* during the COVID-19 outbreak had elevated ALT levels compared to those who did not consume this herbal medicine (Kaewdech et al., 2022). Therefore, ALT elevation should be monitored following the oral administration of high doses of *A. paniculata*. Taken together, there was no significant difference in the frequency of increased AST and ALT levels between the two groups of volunteers who received high doses of andrographolide. Based on this finding, the study suggests that alterations in liver enzymes related to andrographolide may not necessarily be dose-dependent.

After oral administration of 180 mg/day of andrographolide, both AST and ALT levels were significantly increased in male and female participants, indicating no substantial gender differences. However, at a dosage of 360 mg/day, gender-specific variations were observed with females exhibiting a significant elevation in ALT levels while no significant difference was found in males. However, the potential effects of gender differences on safety evaluation should be further investigated in a study with a larger sample size. Despite the mild increase in ALT levels observed in some participants, all of them were able to tolerate these changes, and their ALT levels could return to the normal baseline within the study period. However, the safety and tolerability data obtained from 12 participants per group may be insufficient to conclude the associations between these adverse effects and the oral administration of high doses of *A. paniculata* aqueous extract capsule (180 or 360 mg/day of andrographolide). Further research with larger sample sizes is needed to fully evaluate the potential impact of *A. paniculata* consumption on liver function.

In order to evaluate the usefulness of using *A. paniculata* during the COVID-19 pandemic, we have investigated two key factors: firstly, the appropriate dosage for administration, and secondly, the duration of consecutive administration without adverse effects or minimal side effects. For the appropriate dosage of orally administered *A. paniculata*, the Thailand National List of Essential Medicines (NLEM) recommended for uses of *A. paniculata* at a high dosage equivalent to 180 mg/day of andrographolide for the treatment of patients with mild to moderate COVID-19. The selection of this dosage is determined by considering the preliminary available evidence at the time of the urgent crisis and preliminary documented clinical experiences. A pilot clinical study conducted in Thailand involved six patients with mild symptoms (Benjaponpitak et al., 2021). Treatment of *A. paniculata* extract at a daily dose equivalent to 180 mg/day of andrographolide in combination with standard supportive therapy for 5 days, significantly reduced the severity of COVID-19-related cough and headache (*p* < 0.05) on days 3 and 5. By day 5, three patients tested negative for COVID-19. A recent retrospective cohort study was conducted to investigate the therapeutic and adverse effects of orally administering *A. paniculata* (calculated as 180 mg/day of andrographolide) in early-stage COVID-19 patients. The findings indicated that apart from providing general supportive care, *A. paniculata* has proven a high efficacy in preventing disease progression, including significantly reduced rates of pneumonia, in nonimmune adult patients with early-stage COVID-19, who do not have comorbidities or are not pregnant (Benjaponpitak et al., 2023).

In consideration of the duration of oral administration, Thai Herbal Pharmacopoeia 2021 (Department of Medical Sciences, 2021) suggested that the duration for orally administering *A. paniculata* as an anti-inflammatory for laryngitis should not exceed 7 days. Furthermore, based on the available evidence, a high dose of *A. paniculata* extract (calculated as 180 mg/day of andrographolide) was used for 5 consecutive days during the COVID-19 pandemic in Thailand. It has been shown to effectively inhibit viral replication, mitigate inflammatory effects, and potentially preventing the progression of COVID-19 severity after administering for a period of 5 days (Benjaponpitak et al., 2021; Benjaponpitak et al., 2023). Initiating the administration of *A. paniculata* extract as early as possible is crucial for achieving favorable therapeutic outcomes.

Based on the assessment of the two key factors (dosage and duration), it is evident that the administration of a high dosage of *A. paniculata* extract, equivalent to 180 mg/day of andrographolide, should be carried out for a period of 5 days. Our study reported an increase in liver enzymes after administration of *A. paniculata* aqueous extract (equivalent to 180 mg/day of andrographolide) for 7 days. Therefore, the use of aqueous extract at this high dose should be less than 7 days, unless further studies have been conducted. Additional research is warranted to support the selection of appropriate dose through the comprehensive pharmacokinetic and pharmacodynamic evaluations. The findings obtained from our safety and pharmacokinetic study have indicated a dose relationship between doubling the dosage and doubling the plasma levels. The use of *A. paniculata* aqueous extract at high dose for the treatment of diseases should be adjusted for patients with specific health conditions, in order to minimize adverse effects and maximize treatment outcomes.

## 5 Conclusion

Single and multiple oral administration of a high dose of *A. paniculata* aqueous extract capsule (calculated as 180 or 360 mg/day of andrographolide) was well tolerated in healthy participants. Adverse events were of mild intensity, infrequent in occurrence, and reversible to the normal baseline. A twofold increased dosage resulted in an approximately twofold increase in the plasma levels of the parent compounds and their conjugated metabolites. Furthermore, the results of the biotransformation through a phase II metabolic pathway not only corroborate the plasma concentration levels of the conjugated forms after oral administration of *A. paniculata* aqueous extract capsules, but also provide insight into the elimination kinetics of these conjugated metabolites. Safety consideration should be given to the individual patient’s pertinent health conditions when using this extract in patients with hepatic or kidney dysfunction. The association between the multiple oral administrations of doubled high doses of 180 and 360 mg/day of andrographolide could provide valuable data for dosage adjustment in patients with comorbidities.

## Data Availability

The raw data supporting the conclusion of this article will be made available by the authors, without undue reservation.

## Abbreviations

AUC, Area under plasma concentration-time curve; ALT, alanine aminotransferase; AST, aspartate aminotransferase; BMI, Body mass index; Cl/F, Apparent total clearance of the test substance removed from plasma after oral administration; C_max_, Maximum plasma concentration; CV, Coefficient of variation; ESI, Electrospray ionization; F, Absolute oral bioavailability; GLP, Good laboratory practice; GMP, Good manufacturing practice; HPLC-DAD, High performance liquid chromatography with photodiode array detection; HPLC-MS/MS, High performance liquid chromatography tandem mass spectrometry; ICH-GCP, International conference on harmonization—good clinical practice; IQR, Interquartile range; IRB, Institutional review board; IS, Internal standard; K_el,_ Elimination rate constant; MRM, Multiple reaction monitoring; PVDF, Polyvinylidene difluoride membrane filter; SD, Standard deviation; SGOT, Glutamic-oxalacetic transaminase; SGPT, Glutamic-pyruvic transaminase; THP, Thai Herbal Pharmacopoeia; T_max_, Time to reach maximum plasma concentration; t_1/2_, Elimination half-life; URTIs, Uncomplicated upper respiratory tract infections; V_d_/F, Apparent volume of distribution after non-intravenous administration; WHO-ICTRP, WHO international clinical trials registry platform.
