# Supplementary material for: Comparative pharmacokinetics and safety evaluation of high dosage regimens of Andrographis paniculata aqueous extract after single and multiple oral administration in healthy participants
Source: Front Pharmacol. 2023 Aug 17;14:1230401. doi: 10.3389/fphar.2023.1230401 (PMC10469688; doi:10.3389/fphar.2023.1230401)
Supplement: Supplementary file 1 [file DataSheet1.PDF]

Supplementary Material

Comparative pharmacokinetics and safety evaluation of high dosage regimens of *Andrographis paniculata* aqueous extract after single and multiple oral administration in healthy participants

Phanit Songvut[a], Nuchanart Rangkadilok[a,b], Nanthanit Pholphana[a], Tawit Suriyo[a,b], Duangchit Panomvana[c], Porranee Puranajoti[c], Jaratluck Akanimanee [a], Jutamaad Satayavivad[a,b*]

[a] Laboratory of Pharmacology, Chulabhorn Research Institute, Bangkok, Thailand

[b] Center of Excellence on Environmental Health and Toxicology (EHT), OPS, MHESI, Thailand

[c] Translational Research Unit, Chulabhorn Research Institute, Bangkok, Thailand

*** Correspondence:**

Assoc. Prof. Jutamaad Satayavivad, Ph.D.

E-mail address: jutamaad@cri.or.th

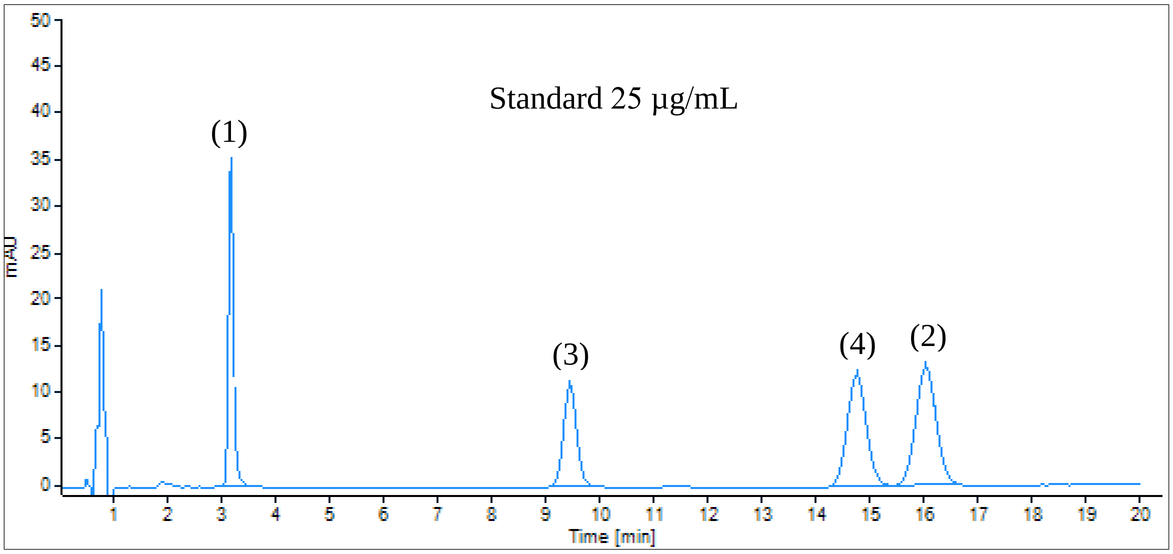

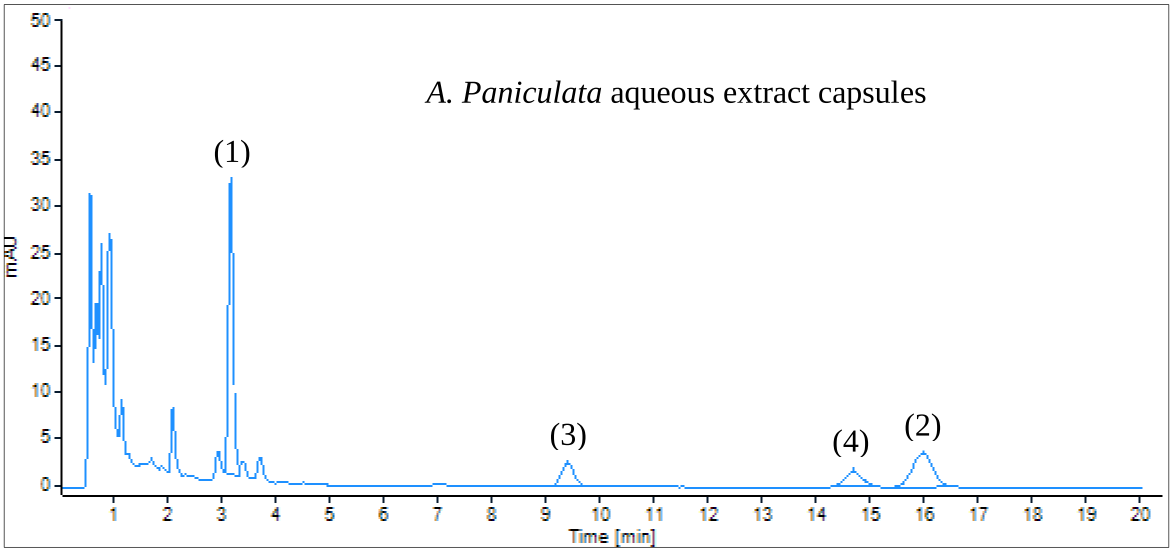

Figure 1S. HPLC chromatograms of the four major standard diterpenoids: **(1)** andrographolide; **(2)**14-deoxy-11, 12-didehydroandrographolide; **(3)** neoandrographolide; **(4)** 14-deoxyandrographolide, and *A. paniculata* aqueous extract capsules.

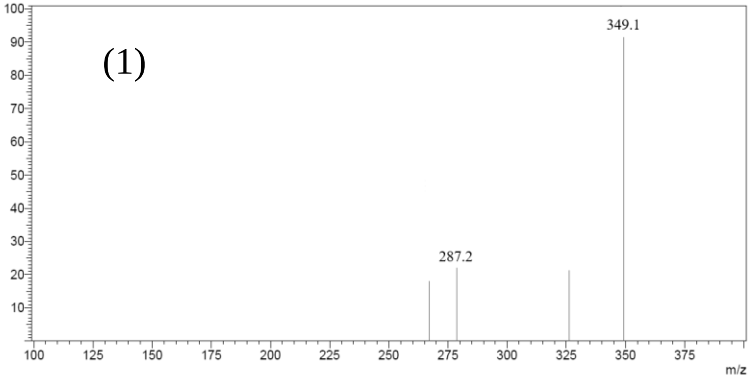

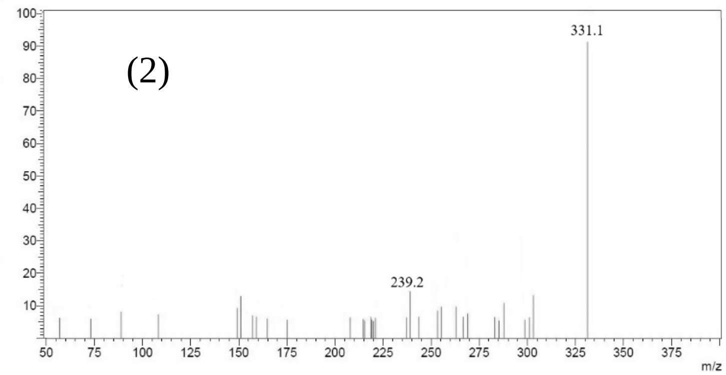

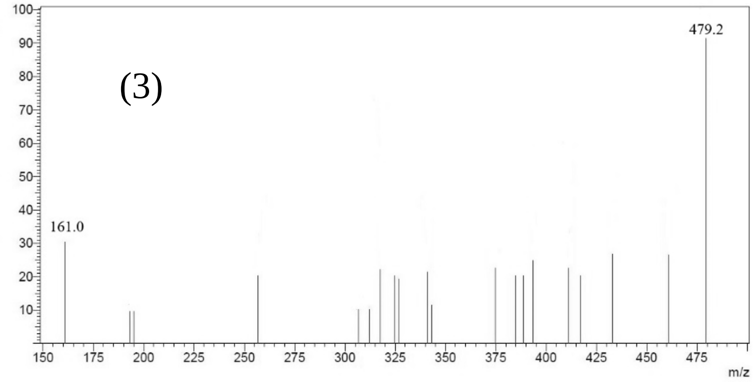

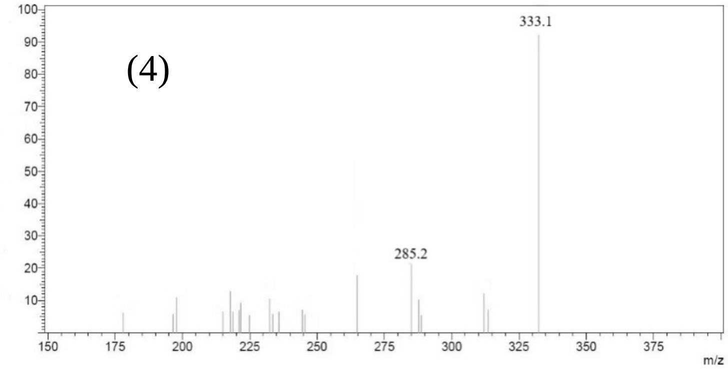

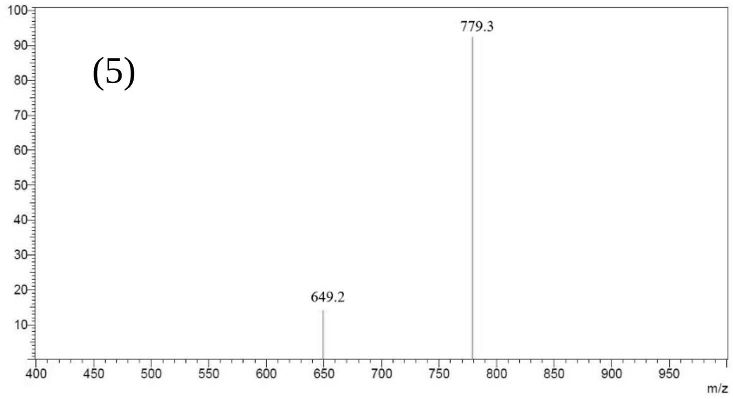

Figure 2S. Mass spectra

(**1**) andrographolide;
(2) 14-deoxy-11,12-didehydroandrographolide;
(3) neoandrographolide;
(4) deoxyandrographolide;
(5) digoxin (IS)

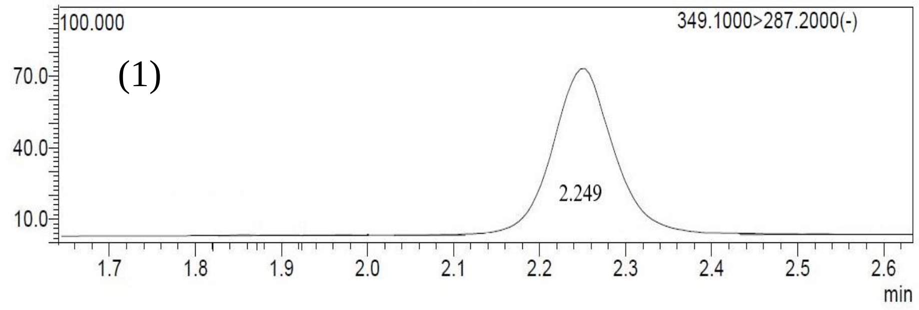

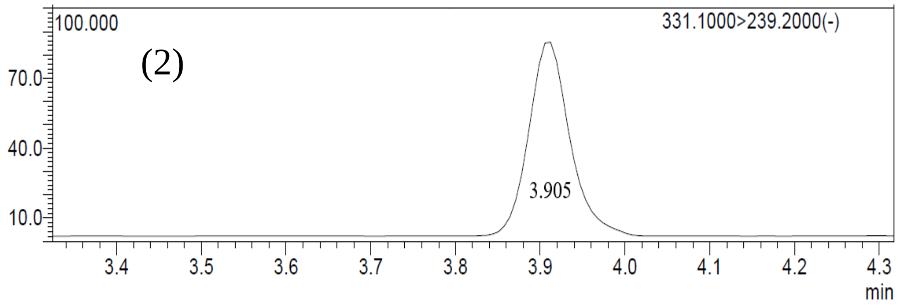

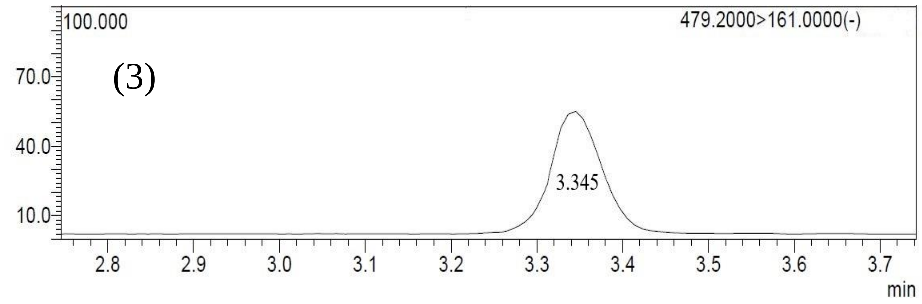

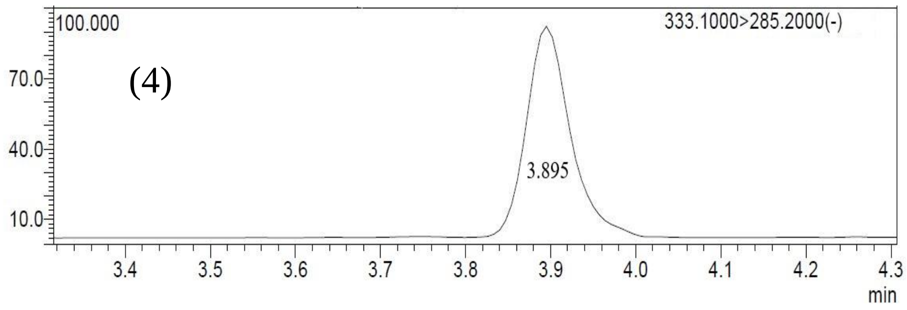

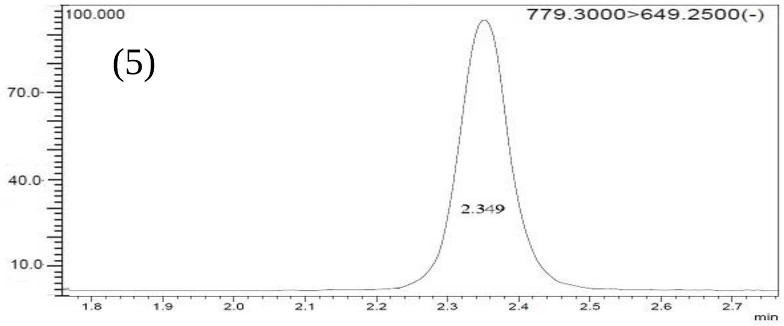

Figure 3S. Chromatograms

(**1**) andrographolide;
(2) 14-deoxy-11,12-didehydroandrographolide;
(3) neoandrographolide;
(4) deoxyandrographolide;
(5) digoxin (IS)
